# Cortical Representations of Transversus Abdominis and Multifidus Muscles Were Discrete in Patients with Chronic Low Back Pain: Evidence Elicited by TMS

**DOI:** 10.1155/2021/6666024

**Published:** 2021-02-18

**Authors:** Xin Li, Howe Liu, Le Ge, Yifeng Yan, Wai Leung Ambrose Lo, Le Li, Chuhuai Wang

**Affiliations:** ^1^Department of Rehabilitation Medicine, The First Affiliated Hospital, Sun Yat-sen University, Guangzhou 510080, China; ^2^Department of Physical Therapy, University of North Texas Health Science Center, Fort Worth, Texas 76101, USA; ^3^Department of Rehabilitation Medicine, The First Affiliated Hospital of Jinan University, Guangzhou 510632, China

## Abstract

**Introduction:**

The transversus abdominis (TVA) and multifidus (MF) muscles are the main segmental spinal stabilizers that are controlled by the primary motor cortex of the brain. However, relocations of the muscle representation in the motor cortex may occur after chronic lower back pain (cLBP); it still needs more evidence to be proven. The current study was aimed at applying transcranial magnetic stimulation (TMS) to investigate the changes of representation of TVA and MF muscles at the cortical network in individuals with cLBP.

**Methods:**

Twenty-four patients with cLBP and 12 age-matched healthy individuals were recruited. Responses of TVA and MF to TMS during muscle contraction were monitored and mapped over the contralateral cortex using a standardized grid cap. Maps of the center of gravity (CoG), area, volume, and latency were analyzed, and the asymmetry index was also computed and compared.

**Results:**

The locations of MF CoG in cLBP individuals were posterior and lateral to the CoG locations in healthy individuals. In the healthy group, the locations of TVA and MF CoG were closed to each other in both the left and right hemispheres. In the cLBP group, these two locations were next to each other in the right hemisphere but discrete in the left hemisphere. In the cLBP group, the cortical motor map of TVA and MF were mutually symmetric in five out of eleven (45.5%) subjects and leftward asymmetric in four out of ten (40.0%) subjects.

**Conclusions:**

Neural representations of TVA and MF muscles were closely organized in both the right and left motor cortices in the healthy group but were discretely organized in the left motor cortex in the cLBP group. This provides strong support for the neural basis of pathokinesiology and clinical treatment of cLBP.

## 1. Introduction

Low back pain (LBP) affects people of all ages and is the third major contributor to the global burden of disability [1]. Pain in the lumbar area with or without accompanying buttock pain over 12 weeks was defined as chronic LBP (cLBP) [2, 3]. The etiology of LBP is complex and involves multiple systems which requires further evidence to clarify the efficacy on interventions for this pathological condition [1]. The evidence that supports structural and functional changes within the central nervous system of people with cLBP is increasing, which appears to play a prominent role in the pathophysiology of these disorders [4, 5]. These neuroplastic changes are reflective of adaptive neurophysiological processes occurring as the result of altered afferent stimuli and cortical areas with cLBP that are initially beneficial, but may persist in a chronic state, and may be part and parcel in the pathophysiology of the condition and the development and maintenance of chronic signs and symptoms [5, 6].

Previous studies suggested that the transversus abdominis (TVA) and multifidus (MF) muscles are the primary segmental spinal stabilization muscles [7] that are controlled by the primary motor cortex of the brain [8, 9]. Understanding how these muscles cooperate to influence lumbopelvic stability is critical to anatomical and biomechanical analysis, as well as to the implementation of effective treatment in patients with LBP. To date, little is known about the relationship between TVA and MF muscles in the cortical representation, which are the local abdominal and back muscles to stabilize the lumbar spine [7]. An anatomical study of the relationship between TVA and MF muscles showed a codependent mechanism that involved a balanced tension between deep abdominal and lumbar spinal muscles, which are linked through the aponeurotic components of the thoracolumbar fascia [10]. A point of equal tension may exist between the MF and TVA muscles [10]. The ability to contract MF was also related to the ability to contract TVA [11], and a poor MF contraction was related to a poor TVA contraction. However, a cross-sectional study found that it was the MF muscle activation, rather than the TVA muscle, that is associated with successful clinical decision using stabilization exercises for patients with cLBP [12]. In addition, some research revealed that individuals with cLBP had increased fatigue of the MF, decreased activation of the TVA [13], and reduced MF muscle thickness at rest and during contraction as compared to healthy individuals [14].

Transcranial magnetic stimulation (TMS) has been used for decades primarily to evaluate changes in the motor cortex in the presence of neurophysiological diseases and to assess therapeutic effects after stimulation [15, 16]. As a noninvasive technique, TMS enables the investigation of the relationship between disorders in the musculoskeletal system and functional changes in the brain, including the adaptive changes of the motor cortex related to the TVA or MF muscles in patients with LBP [2, 3, 17, 18]. The center of gravity (CoG) is known as a robust measure of motor cortical representation and corresponds closely to the area of high excitability of corticomotor neurons that project to the target muscle(s) [19]. A study found that the CoG of the motor cortical mapping of TVA was approximately 2 cm anterior and lateral to the vertex in the healthy group, but the CoG in the LBP group was more posterior and lateral to the CoG location in the healthy group [2]. Another result among healthy people showed that the motor cortical representation for MF was located posterior to that for the erector spinae [3]. In patients with LBP, the short-interval intracortical inhibition level was lower in the left hemisphere and MF volitional contraction was not related to motor cortex excitability [17]. These findings provided preliminary evidence of the reorganization of deep abdominal and lumbar spinal muscle representation in the motor cortex [3, 17, 18], which indicates that using TMS to evaluate the relationship between cortical reorganization and changes in trunk stabilizing muscles does offer a unique insight into building links between the brain (control subsystem), muscles (active musculoskeletal subsystem), and LBP (pain).

Previous studies of TVA and MF muscles' function in the presence of cLBP focused mostly on electromyography (EMG) signals and muscle size in isolation [13, 14], and the studies of the relationship between the two muscles have been limited to the clinical qualitative outcome. Therefore, the first purpose of this study was to compare the cortical motor representation of the TVA and MF muscles in healthy individuals and cLBP patients. The second purpose of this study was to find the relatively changed relationship between TVA and MF in the motor cortex in individuals with and without cLBP. We hypothesized that the CoGs of the cortical motor representation of the two muscles were different between patients with cLBP and healthy individuals and that patients with low back pain have a more discrete relation between the two muscles when compared with healthy individuals. TMS is capable in identifying changes in cortical motor representation; thus, the study protocol was adequately designed to investigate the relationship between the two muscles at the cortical level.

## 2. Materials and Methods

### 2.1. Subjects

Subjects were recruited from the local rehabilitation ward and outpatient department of the hospital. The inclusion criteria for cLBP subjects were as follows: (1) pain in the lumbar area with or without accompanying buttock pain over the past three months [2], (2) pain intensity (perceived during the week preceding the experiment and at the end of experiment) assessed by the 0–10 Numerical Pain Rating Scale (NPRS) with a score ranging from 3 to 7 [20], and (3) ability to perform the experiment procedure. Exclusion criteria were (1) the active existence of respiratory, orthopaedic, circulatory, nephrological, or neurological dysfunctions; (2) previous surgery to the abdomen or lower back; (3) female subjects who were pregnant or suffered from dysmenorrhea; and (4) epilepsy or a family history of epilepsy.

The study protocol was approved by the Medical Ethical Committee of the First Affiliated Hospital, Sun Yat-sen University ([2017]250), and all subjects provided written informed consent before the experiment. The study was conducted in accordance to the Declaration of Helsinki.

### 2.2. Transcranial Magnetic Stimulation

A 7 cm figure-of-eight coil, connected to a Rui Chi magnetic stimulator (Yiruide CCY-IA, Wuhan, China) with a maximum stimulator output (MSO) of 2.0 T, was used to map the neuronal networks of the motor cortex associated with excitation of the contralateral TVA and MF muscles. The figure-of-eight coil provides a better focality of stimulation compared to the standard circular coil and is more ideal for mapping of the motor cortex, as it would evoke fewer ipsilateral responses [2]. The stimulating coil was placed in a crossover position over standardized scalp grids, with the coil handle pointing backward and laterally 45° away from the anterior-posterior axis [2, 3].

### 2.3. EMG Recordings

EMG responses of TVA and MF muscles were detected with two surface electrodes (Ag/AgCl discs, interelectrode distance 2.0 cm, Noraxon, USA). In the healthy control group, bilateral TVA and MF muscles were selected. In the cLBP group, the TVA and MF of the more painful side were selected. The locations of the EMG electrodes were determined in accordance with EMG placement guidelines [21] and published studies [2, 17, 22]. All electrodes were connected to an EMG recording system (Yiruide, Wuhan, China). The motor-evoked potential (MEP) recordings were digitized with a sample rate of 100 kHz, amplified and filtered with a bandpass of 2~10 kHz and a noise eliminator of 50 Hz, and then stored for offline analysis.

### 2.4. Pressure Biofeedback Unit

The Pressure Biofeedback Unit (PBU) (Chattanooga Group Inc., LLC, Vista, California, USA) employed in this study is a widely used noninvasive device for the monitoring of the contraction status of the TVA and MF muscles in patients with LBP in a seated position [22]. Our previous study showed that when the pressure reached 50 mmHg in the seated position, the target for the voluntary contraction of MF and TVA could reach 11.55% MVC (maximum voluntary contraction) and 13.75% MVC, respectively [22]. The pressure of 50 mmHg was a comfortable level for subjects to maintain and minimized the potential of fatigue. The target pressure was displayed on a monitor and the real-time feedback of the pressure was shown to the subject.

### 2.5. TMS Testing of MF and TVA Muscles

**Figure 1:**
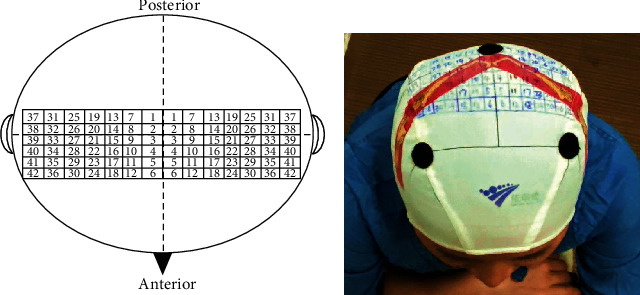
The standard 1 × 1 cm grid cap. (a) Schematic diagram. L: 6 × 7 cm grid; R: 6 × 7 cm grid. (b) Rendering figure. The red band is the primary motor cortex. The two black dots on the ventral side are the left and right dorsolateral frontal lobes. The black dot on the dorsal side is at 1 cm behind the central zero point.

A grid cap was precreated on the sculpture head model [2]. It was a 6 × 7 cm grid system over each hemisphere, from the midline to 6 cm lateral to the vertex and from 2 cm posterior to 5 cm anterior of the vertex [2, 17]. Each standard 1 × 1 cm scalp grid box was numbered to record data and make topographic maps (Figure 1). The inter- and intraexaminer validity of the grid cap mapping system was reported to be excellent [23].

Subjects sat comfortably upright against the chair with their arms well supported on their legs and both feet resting flat on the floor. A tight-fitting standardized scalp elastic cap was worn over the head [24]. The skin was cleaned, and the electrodes were placed at the position in accordance with published studies [2, 17, 22]. The ground pole was connected at the ipsilateral wrist of the measured muscle. To locate the optimal stimulus site for the left or right TVA or MF, the contralateral motor cortex around the anatomical cortex area was stimulated from a single pulse with 70% of the maximum stimulator output (MOS) [2, 25]. The site at which TMS consistently elicited the largest MEPs was determined as the TVA or MF “hot spot.” We took 70% MOS as the baseline, then increased or decreased by 5% MOS increments until an intensity was found that evoked reliable MEPs (≥50 *μ*V in amplitude) in at least five of 10 consecutive trials. The lowest stimulus intensity was determined as the resting motor threshold (RMT) [2, 25].

After RMT was determined, the coil intensity was set to 120% RMT. Subjects were asked to push the pressure cell up to 50 mmHg. Random stimulation was delivered over each point on the cap grid in order to avoid selective bias. Ten stimuli were delivered at each 1 × 1 cm grid at a pressure of 50 mmHg with an interstimulus interval of at least 5 s [2, 3, 17]. We stimulated the contralateral motor cortex of the target muscle. The order of hemispheric stimulation (right or left) was randomized with an equal number of stimulation sequences within the healthy group. An interval of 48 hours was placed between the stimulation of each hemisphere to avoid the interaction effect of TMS on either hemisphere. In patients with cLBP, the contralateral motor cortex of the more painful side was examined. EMG responses from each grid and muscle group were recorded. A point was marked positive when at least five of 10 stimulations evoked reliable MEP (≥50 *μ*V in amplitude). Grid points were stimulated outward from the center until a positive area was demarcated by negative points (MEP < 50 *μ*V). MEP data were stored for offline analysis. This study uses the method of Tsao et al. and the method description partly reproduces their wording [2, 3].

### 2.6. Data Analysis

MEP amplitude was defined as the peak-to-peak voltage of the EMG responses. Five TVA and MF MEPs were averaged at each scalp site. A topographical map of the amplitude of the responses of each muscle was produced by superimposing the MEPs over the respective scalp sites. All responses were normalized to the amplitude of the peak response. Normalized values below 25% of the peak response were removed [2, 17].

Three parameters of the map were calculated from the normalized maps. Map volume, a measure of the total excitability of cortical representation, was calculated as the sum of normalized MEP amplitudes recorded at all scalp sites where responses were evoked. CoG location of the map was calculated using the formula Equation (1), where *x*_*i*_ and *y*_*i*_ are medial-lateral and anterior-posterior locations and *z*_*i*_ is the normalized amplitude [2, 3, 17]. This measure gives an amplitude weighted indication of the map position [2, 3, 17]. The map area was identified as the sites on the scalp grid over each hemisphere from which an EMG response was obtained.

(1) $$\begin{array}{c} \text{CoG}=\frac{\Sigma z_{\dot{i}}x_{i}}{\Sigma z_{i}},\frac{\Sigma z_{i}y_{i}}{\Sigma z_{i}}. \end{array}$$

Latency was defined as the interval from stimulus onset to the individual muscle EMG response. The mean value of the five shortest latencies for each muscle was calculated for analysis [26]. The asymmetry index (AI) was calculated according to the formula Equation (2); *L* and *R* represent the map area covering the left and right hemispheres, respectively [27]. Functional hemispheric lateralization was defined as follows: AI > 0.10 as left-sided lateralization, AI < −0.10 as right-sided lateralization, and −0.10 ≤ AI ≤ 0.10 as mutual lateralization [27]. Python 3.7 (Spyder) was used to create the 3-D plots of the representative maps.

(2) $$\begin{array}{c} \text{AI}=\frac{L-R}{L+R}. \end{array}$$

### 2.7. Statistical Analysis

Statistical analysis was conducted using the SPSS 25.0 software (SPSS Inc., Chicago, IL, USA). Values of dependent variables in each group were described in mean and standard deviations. The Kolmogorov-Smirnov test was used to test the distribution normality of the data. All the variables were normally distributed (*P* > 0.05). Then, a two-way ANOVA was performed to compare the effects of Group (healthy and cLBP) and Side (left and right) of each muscle to assess map volume, area, CoG location (medial-lateral locations and anterior-posterior locations), and latency. If the main effect of the groups was significant, an independent sample *t*-test was conducted to compare the differences between two groups or between two sides. A significant level was set at *P* < 0.05.

## 3. Results

Twenty-four right-handed individuals with cLBP and 12 right-handed healthy individuals with no history of LBP were recruited (Table 1). There was no group difference in gender, age, height, weight, BMI, and educational level (*P* > 0.05). TVA's MEPs could not be evoked over one hemisphere in one healthy subject and one subject with cLBP. The MEPs of MF could not be evoked over one hemisphere in one healthy subject and two subjects with cLBP when using a stimulation intensity of MSO (RMT > MSO).

### 3.1. TMS Mapping

**Figure 2:**
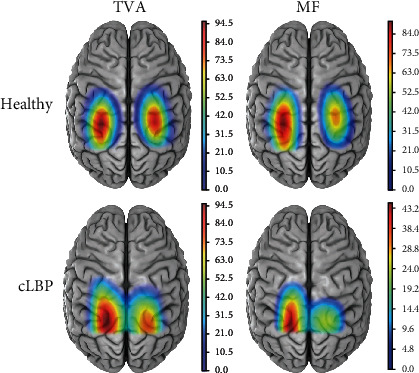
Average representative location of TVA and MF responses to TMS for the healthy and cLBP groups on the left and right hemispheres. cLBP: chronic low back pain; L: left; R: right; TVA: transversus abdominis; MF: multifidus; CoG: center of gravity. Note: this figure was generated from the average data of all the patients. The posterior aspect of the map appeared to be not recorded fully over the entire cortical representation of the muscle. This was due to two cortical motor maps of cLBP patients located more posteriorly. The CoG of TVA and MF from all other participants' muscles in cLBP patients were within the region of the grid cap.

**Figure 3:**
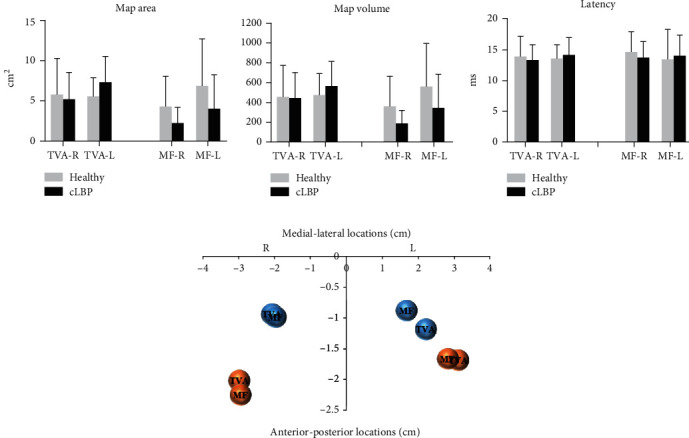
(a–c) Map area, map volume, and latency of TVA and MF MEP responses to TMS for the healthy and cLBP groups on the left and right hemispheres. (d) The relationship between TVA and MF in the left and right hemispheres of healthy and cLBP groups. The blue bubbles denote the CoGs of the muscles in the cLBP group, and the orange bubbles denote the CoGs of the muscles in the healthy group. cLBP: chronic low back pain; L: left; R: right; TVA: transversus abdominis; MF: multifidus; CoG: center of gravity.

Figure 2 shows the average normalized motor cortical representation maps of TVA and MF responses to TMS for the healthy and cLBP groups in the left and right hemispheres. Both the locations of TVA and MF CoG for cLBP were located posterior and lateral to the CoG location in healthy individuals in the left and right hemispheres. For the TVA muscle, there was no statistically significant Group∗Side interaction effect (map area: *F*_interaction_(1, 38) = 0.965, *P* = 0.332; map volume: *F*_interaction_(1, 38) = 0.401, *P* = 0.530; latency: *F*_interaction_(1, 38) = 0.147, *P* = 0.704), the main effect of Group was not significant (map area: *F*_group_(1, 38) = 0.023, *P* = 0.880; map volume: *F*_group_(1, 38) = 0.017, *P* = 0.896; latency: *F*_group_(1, 38) = 0.008, *P* = 0.929), and the main effect of Side was also not significant (map area: *F*_side_(1, 38) = 0.634, *P* = 0.431; map volume: *F*_side_(1, 38) = 0.678, *P* = 0.415; latency: *F*_side_(1, 38) = 0.005, *P* = 0.945). Similar results were found in the MF muscle in that no significant interaction effect or main effects were revealed (map area: *F*_interaction_(1, 34) = 0.497, *P* = 0.485; *F*_group_(1, 34) = 1.811, *P* = 0.187; *F*_side_(1, 34) = 1.099, *P* = 0.302; map volume: *F*_interaction_(1, 34) = 0.218, *P* = 0.644; *F*_group_(1, 34) = 2.457, *P* = 0.126; *F*_side_(1, 34) = 1.758, *P* = 0.194; latency: *F*_interaction_(1, 34) = 0.498, *P* = 0.485; *F*_group_(1, 34) = 0.017, *P* = 0.896; *F*_side_(1, 34) = 0.171, *P* = 0.682) (Figures 3(a)–3(c)).

### 3.2. The Relationship between TVA and MF in the Motor Cortex

Figure 3(d) shows the relationship between TVA and MF in the left and right hemispheres of the healthy and cLBP groups. In the healthy group, the CoGs of the TVA and MF muscles are closed to each other in both the left and right hemispheres. In the cLBP group, they are closed at the right hemisphere, whereas at the left hemisphere, they are obviously discrete. For the TVA muscle, there was no statistically significant Group∗Side interaction effect (maps CoG of the medial-lateral coordinates: *F*_interaction_(1, 38) = 0.020, *P* = 0.889; maps CoG of the anterior-posterior coordinates: *F*_interaction_(1, 38) = 0.900, *P* = 0.349) and the main effect of Side was not significant either (maps of CoG of the medial-lateral coordinates: *F*_side_(1, 38) = 1.497, *P* = 0.229; maps of CoG of the anterior-posterior coordinates: *F*_side_(1, 38) = 0.732, *P* = 0.397). It is worth noting that a significant main effect of Group was found (maps of CoG of the medial-lateral coordinates: *F*_group_(1, 38) = 12.267, *P* = 0.001; maps of CoG of the anterior-posterior coordinates: *F*_group_(1, 38) = 16.121, *P* < 0.0001).

For the MF muscle, the results of two-way ANOVA tests indicated no statistically significant Group∗Side interaction effect (maps of CoG of the medial-lateral coordinates: *F*_interaction_(1, 34) = 2.740, *P* = 0.107; maps of CoG of the anterior-posterior coordinates: *F*_interaction_(1, 34) = 0.026, *P* = 0.872). A significant main effect of Side was found (maps of CoG of the medial-lateral coordinates: *F*_side_(1, 34) = 4.846, *P* = 0.035; maps of CoG of the anterior-posterior coordinates: *F*_side_(1, 34) = 4.762, *P* = 0.036). Again, a significant main effect of Group was found (maps of CoG of the medial-lateral coordinates: *F*_group_(1, 34) = 9.340, *P* = 0.004; maps of CoG of the anterior-posterior coordinates: *F*_group_(1, 34) = 25.019, *P* < 0.0001). Both of the TVA and MF maps of CoG of the medial-lateral and anterior-posterior coordinates show a significant difference in the healthy and cLBP groups (*P* < 0.05) except the MF on the right side of the medial-lateral locations (*P* = 0.420) (Table 2).

### 3.3. Interhemispheric Asymmetry of TVA and MF Muscles

Interhemispheric asymmetry was found in the two groups (Table 3). In the healthy group, both the cortical motor maps of TVA and MF were leftward asymmetric in five out of eleven (45.5%) subjects. In the cLBP group, the cortical motor map of TVA was mutually symmetric in five out of eleven (45.5%) subjects while the cortical motor map of MF was leftward asymmetric in four out of ten (40.0%) subjects.

## 4. Discussion

This study applied TMS to investigate the changes of representation of TVA and MF muscles at the cortical network in individuals with and without cLBP. The results showed discrete organization for the representation of TVA and MF muscles in the left motor cortex and are mutually symmetrical in the cLBP group. Meanwhile, the representation of TVA and MF muscles is closely organized in the right and left motor cortices and is leftward asymmetric in the healthy group. We also found that both the locations of TVA and MF CoGs for cLBP were located posteriorly and laterally compared to those CoG locations observed in healthy individuals. These novel findings revealed the differences between TVA and MF muscle representations in the motor cortex between healthy and cLBP groups.

### 4.1. TVA and MF Muscles Reorganized in the Motor Cortex in Subjects with cLBP

Our results showed that in healthy individuals, TVA representation was located at 1.91 (0.48) cm anteroposterior in the right hemisphere and 1.63 (0.40) cm anteroposterior in the left hemisphere (Figure 2). However, in cLBP individuals, TVA representation was located at 1.14 (0.63) cm anteroposterior in the right hemisphere and 1.15 (0.47) cm anteroposterior in the left hemisphere (Figure 2). The results showed that the motor cortical map of TVA in the LBP group was more posterior and lateral than that of the healthy group, which was supported by the findings of Tsao et al. [2]. Another study by Tsao et al. [3] reported that MF representation was located at 2.6 (0.3) cm mediolateral and 1.4 (0.4) cm anterior to the vertex in healthy individuals, but MF representation has not been studied in individuals with cLBP. However, our results found that the locations of MF CoG were posterior and lateral in individuals with cLBP compared to healthy individuals at the left and right hemispheres. Moreover, the shift was consistently observed in most individuals with cLBP. Our study, together with other early literatures [2, 3], suggested that the present findings were unlikely to be related to cap displacement or inaccurate identification of the vertex.

### 4.2. The Relationship between TVA and MF in the Motor Cortex

Our study revealed that the representation between TVA and MF muscles in the left motor cortex was discrete in subjects with cLBP and close to each other in healthy people (Figure 3(d)). Structural relationships from TMS maps could imply changes in the structural or functional organization of cortical networks that are associated with the activation of TVA and MF muscles in the motor cortex [28]. Evidence of close organization between TVA and MF muscles adds weight to the notion that these muscles may play distinct roles in the control of spinal posture and movement in healthy people [2, 17]. With all of these results, it is reasonable to speculate that the cocontraction of TVA and MF muscles maintains lumbar stability, and this cocontraction diminished in people with cLBP [11, 18]. Interestingly, the present study did not find discrete organization for the representation of TVA and MF muscles at the right hemisphere in the cLBP group. It might be related to the fact that all subjects were right-handed. van den Berg et al. [29] tested right-handers and left-handers with TMS, and the results showed that for right-handed participants, more disruptions were induced when TMS was applied over the left M1 region. Other studies also reported that the left hemisphere was associated with the construction and storage of motor programs, monitoring and modification of movements, and selection and retrieval of motor programs for sequential movements [30–32]. Therefore, we inferred that right-handed cLBP subjects might preferentially recruit their right-sided muscles in performing complex functional tasks, resulting in the TVA and MF muscles being discrete in the representative areas of the left cerebral hemisphere. Additional research is warranted to investigate cortical representation recruitment of left-handed cLBP subjects.

In addition, our results showed that the main reason for the relatively discrete change between the representation of two muscles in the cLBP group was that the shift of the MF muscle was farther away than that of the TVA muscle. This may suggest that the MF muscle disorder in cLBP patients is more prominent than that of TVA. Two early studies [12, 33] reported the clinical importance of the prescription of MF muscle activation for patients with LBP, instead of TVA muscle activation as part of the core stability exercise program. Additionally, atrophic changes of MF were reported in about 77–80% of LBP cases, especially at the L5–S1 level [34], which is the same EMG site of MF in our study. The current study revealed only the changes in the representation of the two muscles in the motor cortex of cLBP patients. Therefore, further studies must link anatomical asymmetries to brain function and behavior using task-related fMRI or ERPs.

### 4.3. Interhemispheric Asymmetry of TVA and MF Muscles

Our study found that there was lateralization to the left (both TVA and MF: 45.5%) in healthy subjects, while there was a trend of mutual symmetry (TVA: 45.5%, MF: 30.0%) in the cLBP groups (Table 3). This hemispheric asymmetry occurs not only in the trunk muscles but also in the swallowing muscles [16], pectoralis major, and latissimus dorsi [34, 35]. Hamdy et al. [16] suggested that there was no consistent relationship between handedness and lateralization. Differences between the left and right hemispheres might be part of a general left-right asymmetry of the motor system in the healthy subjects [36] and might be dependent on the same repetitive event or factors that break body asymmetry. The asymmetry is potentially the neural basis of pathokinesiology of cLBP [35].

### 4.4. Limitations

Despite our important observations, our study had some limitations. Firstly, the subjects in the two groups tended to be younger (mean 28 years). Changes in brain parenchyma are associated with age [37]. Additional studies can expand the sample size and classify age-related differences to investigate the excitation of trunk muscle cortical representations. Moreover, the present study did not consider left-handed subjects, and the current results cannot be applied to left-handed individuals. Future studies are recommended to investigate the relationship between TVA and MF muscles in the motor cortex in left-handed individuals. Lastly, our conclusion is based only on evidence from TMS, but other brain imaging techniques (such as fMRI and EEG) have not yet found it. In future studies, we will further study the brain network relationship between TVA and MF muscles by combining brain function and behavior with task-related fMRI or EEG [38, 39]. The present study adopted the figure-of-eight coil to provide a focalized stimulation on the contralateral motor cortex to map the trunk muscles responses. However, other scholars proposed that the double-corn coil may be more appropriate since it induces contralateral and ipsilateral responses of the trunk muscles consistently [40]. Further research may be warranted to compare the muscle responses elicited by the two different types of coil.

## 5. Conclusions

The cortical representations of TVA and MF muscles were closely organized in the right and left motor cortices and leftward asymmetric in the healthy group. However, the cortical representations of TVA and MF muscles were discretely organized in the left motor cortex and mutually symmetrical in the cLBP group. Brain mapping is fundamental to the understanding of brain organization and function in cLBP patients. Our findings might reveal the relationship between cLBP and cortical reorganization and muscular system dysfunction, providing additional support for the neural basis of pathokinesiology and clinical treatment of cLBP.

## Figures and Tables

**Table 1:** Characteristics of the sample cohorts (mean ± (SD)).

Demographics	cLBP (*n* = 24)	Healthy (*n* = 12)	*P* value
Gender (M : F)	11 : 13	6 : 6	—
Age (years)	28.75 (4.13)	28.17 (4.00)	0.69
Height (cm)	167.92 (9.02)	167.83 (8.08)	0.98
Weight (kg)	61.13 (11.13)	60.75 (9.12)	0.92
BMI (kg/m^2^)	21.48 (2.03)	21.44 (1.89)	0.89
Education level (years)	18.96 (2.63)	20.08 (2.83)	0.27
Side of pain (L : R)	12 : 12	—	—
Pain intensity (NPRS)	4.63 (1.13)	—	—
Pain duration (years)	2.44 (1.72)	—	—
ODI (%)	20.29 (9.32)	—	—

cLBP: chronic low back pain; BMI: body mass index; ODI: Oswestry Disability Index; L: left; R: right; NPRS: numerical pain rating scale; SD: standard deviation.

**Table 2:** The CoG of the medial-lateral (x-CoG) and anterior-posterior coordinates (y-CoG) in the left and right hemispheres of healthy and cLBP groups (mean ± (SD)).

	cLBP	Healthy	*P* value
Medial-lateral locations (cm)			
TVA-R	2.14 (0.56)	2.76 (0.44)	0.011
TVA-L	2.34 (0.84)	3.01 (0.45)	0.035
MF-R	2.64 (0.97)	2.92 (0.30)	0.420
MF-L	1.88 (0.45)	2.81 (0.47)	<0.0001

Anterior-posterior locations (cm)			
TVA-R	1.14 (0.63)	1.91 (0.48)	0.005
TVA-L	1.15 (0.47)	1.63 (0.40)	0.022
MF-R	1.18 (0.60)	2.14 (0.66)	0.004
MF-L	0.72 (0.70)	1.74 (0.42)	0.001

cLBP: chronic low back pain; L: left; R: right; TVA: transversus abdominis; MF: multifidus; CoG: center of gravity.

**Table 3:** TVA and MF muscle dominant hemispheres in healthy and cLBP subjects.

Subjects	Healthy TVA	Healthy MF	cLBP TVA	cLBP MF
1	-0.47	0.09	0.08	-0.56
2	0.38	0.71	0.38	0.33
3	0.60	-0.60	0.23	0.86
4	-0.47	0.00	0.07	0.00
5	-0.50	0.20	0.45	0.00
6	0.08	-0.43	0.00	-0.20
7	-0.22	0.33	0.85	0.25
8	0.43	0.78	0.08	0.64
9	0.43	0.00	-0.24	0.00
10	0.25	0.09	-0.33	-0.65
11	-0.47	0.71	0.00	/
Right-sided (%)	36.4	18.2	18.2	30.0
Left-sided (%)	45.5	45.5	36.4	40.0
Mutual (%)	18.2	36.4	45.5	30.0

cLBP: chronic low back pain; TVA: transversus abdominis; MF: multifidus.

## Data Availability

The datasets generated and analyzed during the current study are available from the corresponding author on reasonable request.
